# Evaluation of the consistence between the results of immunoinformatics predictions and real-world animal experiments of a new tuberculosis vaccine MP3RT

**DOI:** 10.3389/fcimb.2022.1047306

**Published:** 2022-11-02

**Authors:** Peng Cheng, Yong Xue, Jie Wang, Zaixing Jia, Liang Wang, Wenping Gong

**Affiliations:** ^1^ Tuberculosis Prevention and Control Key Laboratory/Beijing Key Laboratory of New Techniques of Tuberculosis Diagnosis and Treatment, Senior Department of Tuberculosis, The 8th Medical Center of PLA General Hospital, Beijing, China; ^2^ Hebei North University, Zhangjiakou, Hebei, China; ^3^ Cangzhou Hospital of Integrated Traditional Chinese and Western Medicine, Cangzhou, Hebei, China

**Keywords:** tuberculosis (TB), vaccine, MP3RT, immune responses, computational tools, immunoinformatics

## Abstract

**Background:**

Our previous study developed a novel peptide-based vaccine, MP3RT, to fight against tuberculosis (TB) infection in a mouse model. However, the consistency between the immunoinformatics predictions and the results of real-world animal experiments on the MP3RT vaccine remains unclear.

**Method:**

In this study, we predicted the antigenicity, immunogenicity, physicochemical parameters, secondary structure, and tertiary structure of MP3RT using bioinformatics technologies. The immune response properties of the MP3RT vaccine were then predicted using the C-ImmSim server. Finally, humanized mice were used to verify the characteristics of the humoral and cellular immune responses induced by the MP3RT vaccine.

**Results:**

MP3RT is a non-toxic and non-allergenic vaccine with an antigenicity index of 0.88 and an immunogenicity index of 0.61, respectively. Our results showed that the MP3RT vaccine contained 53.36% α-helix in the secondary structure, and the favored region accounted for 98.22% in the optimized tertiary structure. The binding affinities of the MP3RT vaccine to the human leukocyte antigen (HLA)-DRB1*01:01 allele, toll-like receptor-2 (TLR-2), and TLR-4 receptors were -1234.1 kcal/mol, -1066.4 kcal/mol, and -1250.4 kcal/mol, respectively. The results of the C-ImmSim server showed that the MP3RT vaccine could stimulate T and B cells to produce immune responses, such as high levels of IgM and IgG antibodies, IFN-γ, TNF-α, and IL-2 cytokines. Results from real-world animal experiments showed that the MP3RT vaccine could stimulate the humanized mice to produce high levels of IgG and IgG2a antibodies and IFN-γ^+^ T lymphocytes. Furthermore, the levels of IFN-γ, IL-2, and IL-6 cytokines in mice immunized with the MP3RT vaccine were significantly higher than those in the control group.

**Conclusion:**

MP3RT is a highly antigenic and immunogenic potential vaccine that can effectively induce Th1-type immune responses *in silico* analysis and animal experiments. This study lays the foundation for evaluating the value of computational tools and immunoinformatic techniques in reverse vaccinology research.

## 1 Introduction

Tuberculosis (TB) is an ancient infectious disease that has continually endangered human life and health. TB still ranks second among the top ten infectious diseases in the world after coronavirus disease 2019 (COVID-19) (Huang and Zhao, 2021; Comas et al., 2013). The Global Tuberculosis Report 2021, released by the World Health Organization (WHO), shows approximately 1.3 million TB deaths in 2020, an increase of roughly 100,000 deaths compared with the previous year (WHO, 2021). Unfortunately, the current situation is still not optimized for the control and prevention of TB.

Vaccination is an effective way to prevent and control TB and other mycobacterial infections. *Mycobacterium bovis* Bacillus Calmette-Guérin (BCG), invented in the 19^th^ century, is the only licensed TB vaccine (Aspatwar et al., 2021). Although BCG has an excellent protective performance on children with severe TB and miliary TB (Mangtani et al., 2014), its effective protection period is only 10-20 years (Andersen and Doherty, 2005). Therefore, there is an urgent need to develop new TB vaccines to make up for the shortcomings of BCG. WHO reported the latest status of the new TB vaccine pipeline in August 2020. There are 14 vaccines in clinical trials, including AEC/BC02, Ad5 Ag85A, ChAdOx185A-MVA85A, MTBVAC, ID93+ GLA-SE, TB/FLU-04L, GamTBvac, DAR-901 booster, H56: IC31, M72/AS01E, BCG revaccination, RUTI^®^, VPM1002, and MIP/Immuvac (WHO, 2020). These vaccines can be divided into four categories: viral vector, protein/adjuvant, inactivated, and attenuated live vaccines. M72/AS01E vaccine is the most promising of these TB vaccines. In 2019, the 3-year final analysis of efficacy, safety, and immunogenicity of the M72/AS01E vaccine was reported, and the results indicated that the vaccine efficacy at month 36 was 49.7%, lower than the 50% efficacy set by the WHO (Tait et al., 2019).

Our previous study developed a novel peptide-based vaccine, MP3RT, and its immunological specificity and protective efficiency were evaluated in humanized and wild-type mice (Gong et al., 2021a). Compared with these traditional TB vaccines mentioned above, peptide-based vaccines have significant advantages, including good antigenicity, efficient recognition by major histocompatibility complex (MHC) molecules, fewer side effects, simple production, and accessible transportation (Bijker et al., 2007; Moyle and Toth, 2008; Gong et al., 2021b; Gong et al., 2022).

The rapid development of computational tools and immunoinformatic techniques provides an opportunity for the rise of reverse vaccinology (Moodley et al., 2022). Immunoinformatics is an interdisciplinary subject based on informatics and modern immunology that has emerged in recent years. The rapid development of immunoinformatics has promoted the progress of contemporary immunology and new vaccine research. It has been widely used for epitope discovery, precise immune engineering, and accelerated vaccine design (De Groot et al., 2020; Moise et al., 2020; Kumar et al., 2020; Kumar et al., 2021). Based on immunoinformatics and molecular biology, reverse vaccinology is developed to screen candidate antigens with protective immune responses from the whole gene level. This strategy has the advantages of convenience, wide range, safety, workforce saving, and cost-saving (Elhag et al., 2019). However, the consistency between the immunoinformatics predictions and the results of real-world animal experiments on the MP3RT vaccine remains unclear. Therefore, this study was performed to predict the antigenicity, immunogenicity, secondary structure, and physicochemical properties of the MP3RT vaccine using multiple bioinformatics tools. In addition, we also analyzed its functional predictions, such as tertiary structure modeling, molecular docking, and immune stimulation. Finally, we conducted animal experiments to verify the MP3RT vaccine’s immunological properties and evaluated the value of bioinformatics technology in reverse vaccinology research by comparing and analyzing real-world data and bioinformatics prediction data.

## 2 Materials and methods

### 2.1 Bioinformatics prediction

#### 2.1.1 MP3RT molecular sequence

In our previous study, the MP3RT vaccine was composed of six immunodominant peptides (Mtb8.4_69-83_, PPE18_115-129_, PPE18_149-163_, PPE68_138-152_, RpfA_377-391_, and TB10.4_21-35_), derived from five antigens including Mtb8.4 (Rv1174c) (Liu et al., 2016; Liu et al., 2017; Bai et al., 2018), PPE18 (Rv1196) (Hebert et al., 2007; Homolka et al., 2016), PPE68 (Rv3873) (Hanif et al., 2010; Christy et al., 2012; Hanif and Mustafa, 2017), RpfA (Rv0867c) (Romano et al., 2012), and TB10.4 (Rv0288) (Hoang et al., 2013; Rashidian et al., 2016; Meshkat et al., 2016). Numerous studies have identified these five antigens as potential TB vaccine candidates (**Table 1**) (Zvi et al., 2008; Gaseitsiwe et al., 2010; Liu et al., 2016; Gong et al., 2021a). In this study, the amino acid sequence of the MP3RT vaccine was obtained from the previous study of Gong, W. et al. (Gong et al., 2021a), as follows: MSDKIIHLTDDSFDTDVLKADGAILVDFWAEWCGPCKMIAPILDEIADEYQGKLTVAKLNIDQNPGTAPKYGIRGIPTLLLFKNGEVAATKVGALSKGQLKEFLDANLAGSGSGHMHHHHHHSSGLVPRGSGMKETAAAKFERQHMDSPDLGTDDDDKAMAMLRNFLAAPPPQRAAMGGGGSRAELMILIATNLLGQGGGGSAAAMFGYAAATATATGGGGSDYFIRMWNQAALAMEGGGGSAYTKKLWQAIRAQDVGGGGSYAGTLQSLGAEIAVEHHHHHH.

**Table 1:** Summary of basic information about the five antigens that make up the MP3RT vaccine (Gong et al., 2021a).

Protein Name	Accession No ^a^	Locus_tag	Gene ^b^	Length (aa)	Annotation ^b^	Summary Information ^b^	References
Mtb8.4	CCP43930	Rv1174c	*TB8.4*	110	Low molecular weight T-cell antigen TB8.4	Predicted to be an outer membrane protein and possible vaccine candidate	(Liu et al., 2016; Liu et al., 2017; Bai et al., 2018)
PPE18	CCP43952	Rv1196	*PPE18*	391	PPE family protein PPE18	Member of the *Mycobacterium tuberculosis* PPE family	(Hebert et al., 2007; Homolka et al., 2016)
PPE68	CCP46702	Rv3873	*PPE68*	368	PPE family protein PPE68	A peptide-based vaccine candidate	(Hanif et al., 2010; Christy et al., 2012; Hanif and Mustafa, 2017)
RpfA	CCP43615	Rv0867c	*rpfA*	407	Possible resuscitation-promoting factor RpfA	Predicted possible vaccine candidate	(Romano et al., 2012)
TB10.4	CCP43018	Rv0288	*esxH/cfp7/TB10.4*	96	Low molecular weight protein antigen 7 EsxH	Predicted possible vaccine candidate	(Hoang et al., 2013; Rashidian et al., 2016; Meshkat et al., 2016)

a The National Center for Biotechnology Information (NCBI, http://www.ncbi.nlm.nih.gov/). Data were retrieved on Aug 12, 2022.

b The Gene name, annotation, and summary information are based on the data deposited at the NCBI. Data were retrieved on Aug 12, 2022.

#### 2.1.2 Immunogenicity, antigenicity, allergenicity, and toxicity prediction of the MP3RT vaccine

The immunogenicity and antigenicity of a vaccine play an essential role in fighting against *M. tuberculosis* infection. Therefore, the immunogenicity of the MP3RT vaccine was predicted using the Immune Epitope Database (IEDB) immunogenicity server (http://tools.iedb.org/immunogenicity/). The immunogenicity of the MP3RT vaccine was determined with a score, and a higher score indicates a greater probability of eliciting an immune response. Subsequently, the antigenicity of the MP3RT vaccine was predicted using VaxiJen v2.0 (http://www.ddg-pharmfac.net/vaxijen/VaxiJen/VaxiJen.html) and analyzed by the ANTIGENpro server (http://scratch.proteomics.ics.uci.edu/) for further verification following previous studies (Doytchinova and Flower, 2007; Magnan et al., 2010). The antigenicity of the MP3RT vaccine was evaluated by a new alignment-free approach based on auto cross covariance (ACC) transformation of protein sequences into uniform vectors of principal amino acid properties, and the prediction accuracy of the VaxiJen model is 70%–89% under the threshold of 0.50 (Doytchinova and Flower, 2007).

To better verify the toxic and side effects of the MP3RT vaccine, AllerTOP v.2.0 server (http://www.ddg-pharmfac.net/AllerTOP/) and Allergen FP v.1.0 server (http://ddg-pharmfac.net/AllergenFP/) were used to predict the sensitivity of the MP3RT (Dimitrov et al., 2014; Dimitrov et al., 2014). Finally, the Toxin Pred server (http://crdd.osdd.net/raghava/toxinpred/) was used to predict the toxicity of the MP3RT vaccine. The prediction results of both the AllerTOP v.2.0 server and Toxin Pred server were shown as non-”Sensitization” or “No-sensitization” and “Non-toxicity” or “Toxicity”, respectively.

#### 2.1.3 Physiochemical properties and solubility of MP3RT

The physicochemical parameters of the MP3RT vaccine were predicted by the ExPASy ProtParam server (https://web.expasy.org/protparam/), including theoretical isoelectric point (pI), *in vitro* and *in vivo* half-life, instability index, and aliphatic index according to a previous study (Wilkins et al., 1999). The theoretical pI of a protein can facilitate the selection of methods for protein purification, such as ion exchange chromatography or isofocusing electrophoresis (Pergande and Cologna, 2017). Half-life refers to the time it takes for half of the synthesized protein to disappear (Ciechanover and Schwartz, 1989). The instability index shows the stability of the protein in the test tube. If the predicted value is less than the threshold of 40, it is considered stable (Guruprasad et al., 1990), and the instability index range of 16.90 to 38.78 suggests that the protein is highly stable (Dutta et al., 2018). The aliphatic index refers to the relative volume occupied by the aliphatic side chains of the protein, which can indicate the thermal stability of the protein (Ikai, 1980). The aliphatic index range of 39.80 to 90.68 suggests that the protein is highly thermostable, and alanine amino acid is ubiquitous in the aliphatic side chain (Dutta et al., 2018). Furthermore, the solubility of the MP3RT vaccine was predicted using the Protein–Sol server (https://protein-sol.manchester.ac.uk/). This server provides a rapid sequence-based method for the detection of protein solubility. Solubility prediction on the server is given in the 0-1 range for ease of user interpretation. When the predicted value is above the threshold of 0.45, it is suggested that a protein has good solubility (Hebditch et al., 2017).

#### 2.1.4 Secondary structure and three-dimensional (3D) structure prediction and refinement

The PSIPRED server (http://bioinf.cs.ucl.ac.uk/psipred/) was used to predict the secondary structure of the MP3RT vaccine. This server uses a specific scoring matrix to predict α-helices, β-sheets, and random coils in protein structures (McGuffin et al., 2000). Next, the 3Dpro server (Scratch Protein Predictor (uci.edu)) was used to predict the 3D spatial arrangement of the MP3RT vaccine. 3Dpro uses predicted structural features and the Protein Data Bank (PDB) knowledge-based statistical terms in the energy function (Cheng et al., 2005). Therefore, 3Dpro is most appropriate to use with targets that do not have good structural templates (Cheng et al., 2005). Subsequently, the initial model was optimized by the GalaxyRefine web server (https://galaxy.seoklab.org/cgi-bin/submit.cgi?type=REFINE), which improved both global and local structure quality on average. This server refines the side chains and performs side-chain re-packing followed by overall structural relaxation through molecular dynamics simulation (Heo et al., 2013).

#### 2.1.5 Tertiary structure and molecular docking

The ERRAT server (https://saves.mbi.ucla.edu/) was used to evaluate the uncertainty of the 3D structure of the MP3RT vaccine. ERRAT server uses characteristic atomic interactions to distinguish appropriate but inaccurately determined regions in 3D models (Colovos and Yeates, 1993). In addition, Ramachandran diagrams were drawn *via* the SWISS-MODEL server (https://swissmodel.expasy.org/assess) (Waterhouse et al., 2018). This server visualizes the percentage of residues in favored, outlier, and rotamer regions based on the backbone dihedral angle (Ф) and (ψ) of each amino acid in the vaccine candidate (Waterhouse et al., 2018).

The PDB files for human leukocyte antigen (HLA)-DRB1*01:01 (ID: 1AQD), toll-like receptor-2 (TLR-2, 6NIG), and TLR-4 (4G8A) receptors were obtained from Molecular Modeling Database (MMDB, https://www.ncbi.nlm.nih.gov/structure/). The PDB file of the MP3RT vaccine was obtained from the GalaxyRefine web server. To evaluate the affinity between the MP3RT vaccine and the HLA-DRB1*01:01 allele, TLR-2, and TLR-4 receptors, the ClusPro2.0 server (https://cluspro.bu.edu/home.php) was used to perform ligand-receptor docking analysis (Kozakov et al., 2017). The ClusPro server simulates molecular docking mainly based on the following algorithms: rigid body docking is accomplished by sampling billions of conformations, clustering of the 1000 lowest energy structures based on root mean square deviation (RMSD), finding the best models, and the selected models are optimized using energy minimization. By default, the server only displays the top 10 models. Finally, the PyMOL2.5.3 software (Schrödinger, New York, USA) was used for visualization analysis.

#### 2.1.6 Prediction of conformational B-cell epitopes

Conformational B-cell epitopes result from protein folding by which distant residues can be brought close to each other to form conformational B-cell epitopes. It is estimated that more than 90% of B cell epitopes are discontinuous. Therefore, we predicted the conformational B-cell epitopes of the MP3RT vaccine through the ElliPro server (http://tools.iedb.org/ellipro/) (Ponomarenko et al., 2008).

#### 2.1.7 Immune simulation

Immune stimulation was performed using the C-ImmSim server (https://150.146.2.1/C-IMMSIM/index.php), which can predict the expression changes of T and B lymphocytes and cytokines after stimulation with the MP3RT vaccine (Rapin et al., 2010). The parameters were set: random seed, simulation volume select default value, HLA select server recommended allele. The simulation step was selected as 800, and 3 injections of the MP3RT vaccine were performed. The injection time was set on days 0, 28, and 42.

### 2.2 Experimental verification

#### 2.2.1 Experimental animals

Female humanized C57BL/6 mice (HLA-A11^+/+^DR1^+/+^H-2-b2m^-/-^/IAb^-/-^) with similar weight and age were gifted by Professor Yusen Zhou of the Beijing Institute of Microbiology and Epidemiology (Beijing, China). The animal experiments were conducted following the Experimental Animal Regulation Ordinances principles established by the China National Science and Technology Commission. The mice were raised with the utmost humanitarian care, and all were put to death under anesthesia to reduce their suffering. Protocols on mice were approved by the Animal Ethical Committee of the 8th Medical Center of PLA General Hospital (Approved Number: 309201808171015).

#### 2.2.2 Mice immunization

The immunization strategy of mice was conducted following our previous study (Gong et al., 2021a). In brief, the humanized mice were divided into two groups. First, the mice in the control group (*n* = 6) were injected with 30 μg CpG oligonucleotide (ODN2395) adjuvant (Sangon, Shanghai, China) in 100 ml phosphate buffer saline (PBS). Next, the mice in the MP3RT group (*n* = 7) were immunized with 30 μg MP3RT combined with 30 μg CpG-ODN2395 adjuvant in 100 ml PBS for the primary immunization. After primary immunization, the mice in the MP3RT group were immunized with 20 μg MP3RT in 100 ml PBS on day 28 and day 42, respectively. The mice in the control group were inoculated with 20 μg GpG-ODN2395 in 100 ml PBS on day 28 and day 42, respectively. Then, mice’s blood samples were collected on days 0, 14, 28, 42, 56, and 70 after the first immunization. Finally, mice were sacrificed on day 84 after the primary vaccination, and the spleens of the mice were collected.

#### 2.2.3 MP3RT-specific antibody detection

The blood sample of each mouse was centrifuged at 2500rpm for 10 min, and the serum was isolated and stored at -80°C;. In order to reduce individual differences, the sera of mice in each group were pooled and tested in quadruplicate for antibody detection, respectively. The MP3RT-specific antibody IgG and its subtypes IgG1 and IgG2a were detected using an indirect Enzyme-Linked Immune Sorbent Assay (ELISA) following our previous study (Gong et al., 2021a).

#### 2.2.4 Enzyme-linked immunospot (ELISPOT) array

On 84 days after primary immunization, mice in MP3RT and PBS groups were killed. The spleen of each mouse was collected and prepared into 10 ml spleen cell suspension according to our previous studies (Wang et al., 2017; Gong et al., 2021a; Gong et al., 2021b). A volume of 100 μl splenocytes with the concentration of 2.5×10^6^/ml was added to each well of the 96-well ELISPOT plate. Then, 50 μl of PBS (as negative control), 50 μl of MP3RT vaccine (60 μg/ml), or 50 μl of phytohemagglutinin (PHA, as positive control, 60 μg/ml) were added into the wells and incubated with splenocytes at 37°C for 24h, respectively. Then, the frequency of interferon-γ (IFN-γ)^+^ T lymphocytes was determined using a Mouse IFN-γ ELISOPT^PLUS^ (Mabtech AB, Nacka Strand, Sweden) following the manufacturer’s instructions. Finally, the spots forming cells (SFCs) were confirmed with a CTL-S5 Versa ELISPOT Reader (CTL, Cleveland, OH, USA). The stimulation index (SI) value was used to represent the ability of the MP3RT vaccine to induce IFN-γ^+^ T lymphocytes. To reduce individual differences, the splenocytes of mice in each group were pooled together and tested in quadruplicate for IFN-γ^+^ T lymphocytes detection.

#### 2.2.5 Cytokines detection

Splenocytes collected from mice were prepared following the above method. Then, cytokines such as interleukin-2 (IL-2), IL-4, IL-6, IL-10, IFN-γ, tumor necrosis factor-α (TNF-α), and IL-17A were detected by a Mouse Th1/Th2/Th17 Cytokine Kit (BD Biosciences, San Jose, CA, USA) following the manufacturer’s instruction. To reduce individual differences, the splenocytes of mice in each group were pooled together and tested in quadruplicate for cytokine detection.

### 2.3 Statistical analysis

The data of MP3RT-specific antibodies, SI, and cytokines were analyzed by using the GraphPad Prism 9.4.0 (San Diego, CA, USA). In brief, the levels of cytokines induced by the MP3RT vaccine were analyzed with the Unpaired t-test or Mann-Whitney test according to the normality. The data were shown as mean ± standard error of the mean (SEM), and *P*-value < 0.05 was considered a significant difference.

## 3 Result

### 3.1 Immunogenicity, antigenicity, allergenicity, and toxicity of the MP3RT vaccine

Our results showed that the immunogenicity and antigenicity index of the MP3RT vaccine predicted by the IEDB Immunogenicity server and VaxiJen v2.0 server were 0.61 and 0.83, respectively. Subsequently, the antigenicity of the MP3RT vaccine was further verified by the ANTIGENpro server and showed a 0.88 value. Furthermore, the predicted results of the AllerTOP v. 2.0 server, Allergen FP v. 1.0 server, and Toxin Pred server indicated that the MP3RT vaccine was non-sensitizing and non-toxic. These data suggest that MP3RT is a potential TB vaccine with high antigenicity, strong immunogenicity, non-toxicity, and non-sensitization.

### 3.2 Physicochemical property and solubility of the MP3RT vaccine

**Figure 1:**
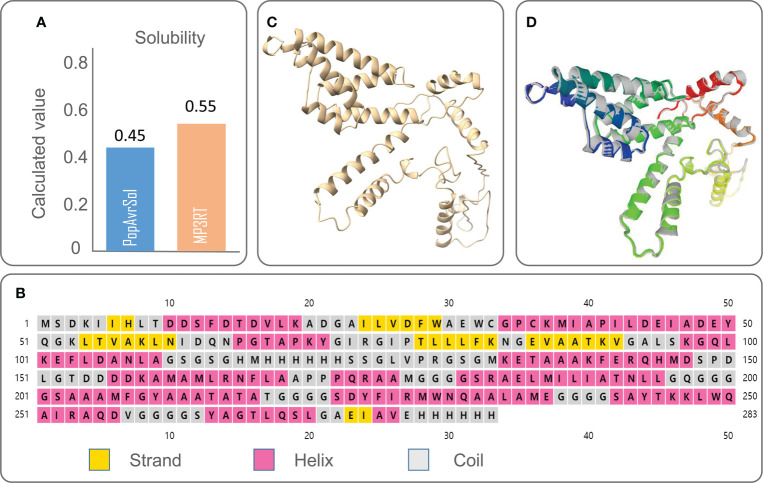
The solubility, secondary structure, tertiary structure, and optimized tertiary structure of the MP3RT vaccine. **(A)** Solubility of the MP3RT vaccine was predicted by the Protein–Sol server. **(B)** Secondary structure of the MP3RT vaccine was predicted by the PSIPRED server. **(C)** The 3Dpro serve was used to predict the tertiary structure of the MP3RT vaccine before optimization. **(D)** The optimized tertiary structure of the MP3RT vaccine was predicted by the Galaxy WEB server. The colored parts in the picture are improvements to all sidechain structures that initialize the model, improving the structure accuracy and physical correctness of the global and partial parts through repeated relaxation.

The physicochemical properties of a vaccine significantly affect the development of immunological functions. We obtained the physicochemical parameters of the MP3RT vaccine through the ExPASy ProtParam server, and the results were shown in **Table 2**. The solubility of the MP3RT vaccine was predicted to be 0.55 by the Protein–Sol server (**Figure 1A**), indicating that this vaccine had good solubility. This expected result is consistent with the experimental result in our previous study (Gong et al., 2021a).

**Table 2:** Physicochemical parameters of the MP3RT vaccine predicted by the ExPASy ProtParam server.

Number of amino acids	Molecular weight (Da)	Theoretical pI	Estimated half-life (Hour)	Instability index^*^	Aliphatic index^#^	GRAVY
283 aa	29640.46	6.08	30 hours (mammalian reticulocytes, *in vitro*). >20 hours (yeast, *in vivo*). >10 hours (Escherichia coli, *in vivo*).	29.65	76.08	-0.21

^*^Instability index is less than the threshold of 40, it is considered stable.

^#^The aliphatic index range of 39.80 to 90.68 indicates that the protein is highly thermostable, and alanine amino acid is ubiquitous in the aliphatic side chain.

### 3.3 Secondary structure, tertiary structure, and tertiary structure validation of MP3RT

According to the PSIPRED server, we predicted the secondary structure of the MP3RT vaccine (**Figure 1B**), and the results showed that this vaccine consisted of 53.36% α-helix, 10.6% β-strand, and 36.04% random coil. The 3D structure of the MP3RT vaccine model was initially designed using the 3Dpro server (**Figure 1C**). Subsequently, the MP3RT vaccine model was optimized using the Galaxy WEB server. The optimized models were ranked by GDT-HA and MolProbity (**Table 3**). The I-TASSER server prediction results include five parameters. GDT-HA represents the accuracy of the backbone structure in which the model was built. RMSD refers to the structural deviation of a moment relative to the reference conformation. MolProbity scores include: the number of atomic clashes per 1000 atoms (clash score), the percentages of rotamer outliers (poor rotamers), and Ramachandran favored backbone torsion angles (Rama favored). Therefore, the MolProbity score provides a broad-spectrum reliable assessment of model quality for proteins and nucleic acids at global and local levels. Therefore, a higher GDT-HA value and a lower MolProbity value indicate a better model quality. Herein, Model 5 (**Figure 1D**) with higher GDT-HA scores and lower MolProbity scores was used for further research.

**Table 3:** The model parameters refined by the Galaxy WEB server.

Model	GDT-HA	RMSD	MolProbity	Clash score	Poor rotamers	Rama favored
Initial	1.0000	0.000	3.521	100.0	5.3	91.5
MODEL 5	0.9161	0.481	1.617	11.8	1.0	97.9
MODEL 1	0.9134	0.493	1.624	13.0	1.0	98.2
MODEL 2	0.9134	0.488	1.677	13.7	0.5	97.9
MODEL 3	0.9214	0.475	1.701	12.3	0.5	97.5
MODEL 4	0.9028	0.509	1.856	14.2	1.0	96.8

### 3.4 Validation of tertiary structure and molecular docking

**Figure 2:**
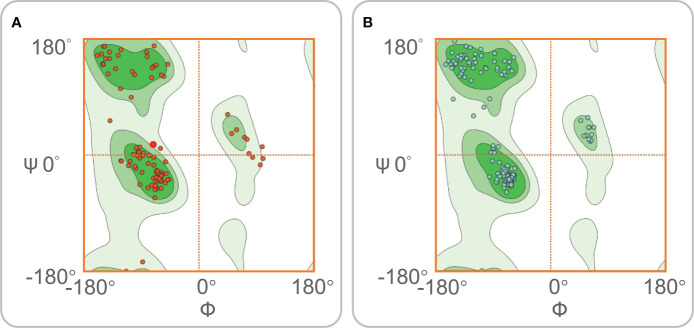
Ramachandran diagrams for the MP3RT vaccine. Ramachandran map is a method of visualizing energy preference regions, which can be used to see if protein structure is reasonable. The dark green area is the favored region. The other two green areas are the outlier region. The white area is the rotamer region. The rotamer region can be interpreted as the high-energy region, and the protein needs to spend some energy to drive the residues into this region. **(A)** Before optimization, the Ramachandran diagram showed that the favored region, outlier region, and rotamer region of the MP3RT vaccine were 91.34%, 3.15%, and 5.32%. **(B)** After optimization, the Ramachandran diagram showed that the favored region, outlier region, and rotamer region of the MP3RT vaccine were 98.22%, 0.36%, and 0.99%, respectively.

**Figure 3:**
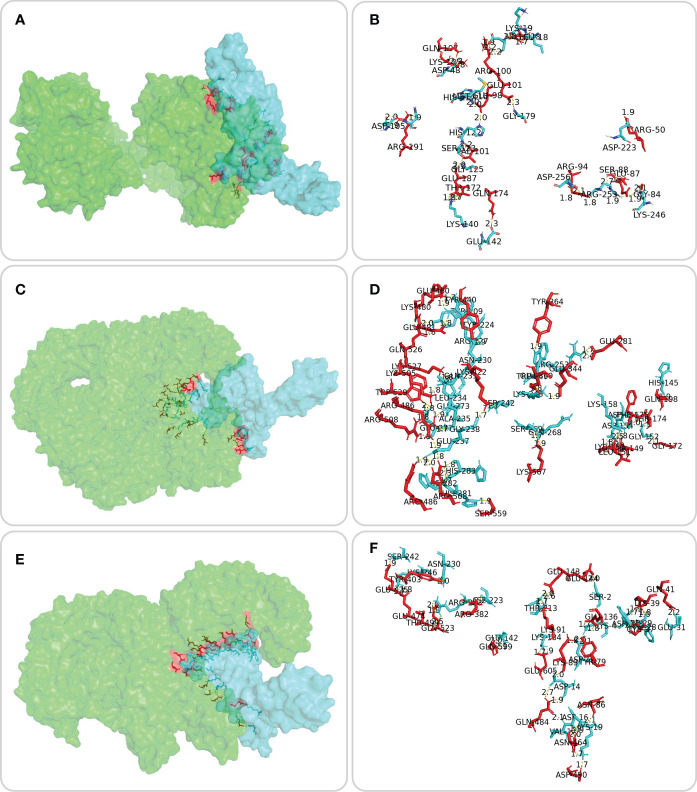
Molecular docking between the MP3RT vaccine and the HLA-DRB1*01:01 molecule, TLR-2, and TLR-4 receptors. Molecular docking of the MP3RT vaccine (blue) with HLA-DRB1*01:01 (**A**, green), TLR2 (**C**, green), and TLR4 (**E**, green) was performed using ClusPro2.0 server (https://cluspro.bu.edu/home.php). PDB files for TLR2 (6NIG) and TLR4 (4G8A) receptors were obtained from the Molecular Modeling Database (MMDB, https://www.ncbi.nlm.nih.gov/structure/). PyMOL2.5.3 was used to visualize and analyze the amino acid sites where the MP3RT vaccine (blue) docked with HLA molecule (**B**, red), TLR2 (**D**, red), and TLR4 (**F**, red). The detailed information of ligand amino acid, distance, and acceptor amino acid for molecular docking of the MP3RT vaccine with HLA-DRB1*01:01, TLR-2, and TLR-4 can be found in **Table S1**, **Table S2**, and **Table S3**, respectively.

The ERRAT server was used to validate the 3D structure model of the MP3RT vaccine. The overall quality factor of the MP3RT vaccine increased from 64.84% to 81.75%. Ramachandran plot analysis also yielded some improvements, the favored region rose from 91.34% to 98.22%, the outlier region reduced from 3.15% to 0.36%, and the rotamer region decreased from 5.32% to 0.99% (**Figures 2A, B**). Furthermore, the ClusPro2.0 server was used to perform ligand-receptor docking analysis. The results showed that the lowest binding energy required for MP3RT vaccine to HLA-DRB1*01:01, TLR-2, and TLR-4 receptors were -1234.10 kcal/mol, -1066.40 kcal/mol, and -1250.40 kcal/mol, respectively. The visualization of ligand-receptor docking between the MP3RT vaccine and HLA-DRB1*01:01, TLR-2, or TLR-4 receptor was shown in **Figure 3**. Furthermore, we assessed the distances between amino acid residues that interact between the HLA-DRB1*01:01 (**Table S1**), TLR-2 (**Table S2**), and TLR-4 (**Table S3**) receptors and the MP3RT vaccine.

### 3.5 Conformational B−cell epitopes

**Figure 4:**
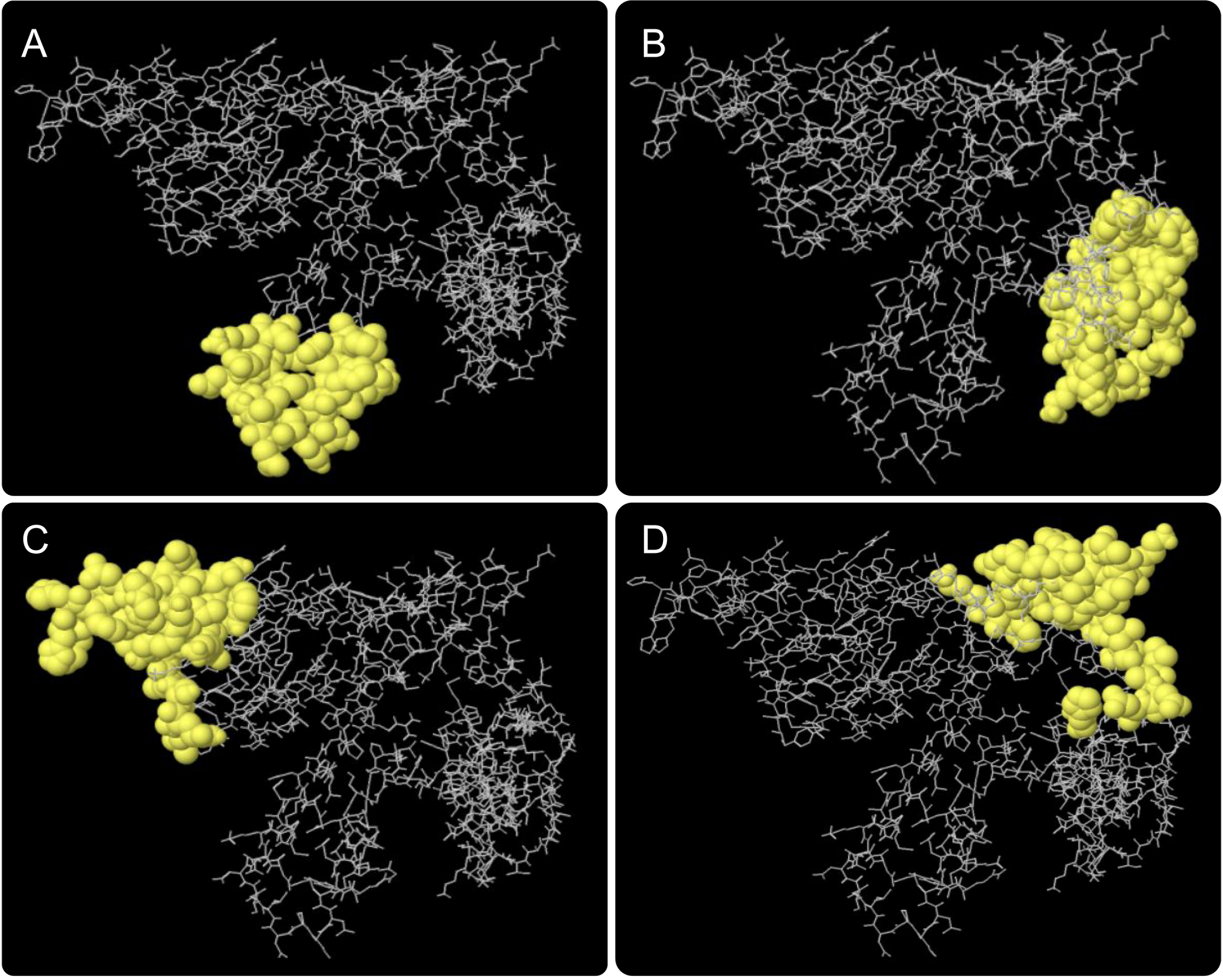
Conformational B cell epitopes predicted by the ElliPro server. The positions of the yellow balls in **(A–D)** indicate the positions of conformational B cell epitopes, and the rest were shown in gray.

Results of the ElliPro server found that a total of four conformational B cell epitopes were formed, and their scores were 0.82 (**Figure 4A**), 0.73 (**Figure 4B**), 0.71 (**Figure 4C**), and 0.67 (**Figure 4D**), respectively. Typically, we select epitopes with a score > 0.69, and the results showed that three conformational B-cell epitopes with a score >0.69 contained 112 residues (**Table 4**).

**Table 4:** The conformational B cell epitopes of the MP3RT vaccine predicted by the ElliPro.

Residues	Number of residues	Score
_:T136, _:A137, _:A138, _:A139, _:K140, _:F141, _:E142, _:R143, _:Q144, _:H145, _:M146, _:D147, _:S148, _:P149, _:D150, _:L151, _:G152, _:T153, _:D154, _:D155, _:D156, _:D157, _:K158, _:A159, _:M160	25	0.82
_:Y50, _:Q51, _:G52, _:K53, _:L54, _:T55, _:V56, _:A57, _:K58, _:L59, _:N60, _:I61, _:D62, _:Q63, _:N64, _:P65, _:G66, _:T67, _:A68, _:P69, _:K70, _:Y71, _:G72, _:I73, _:R74, _:G75, _:I76, _:P77, _:T78, _:L79, _:L80, _:L81, _:F82, _:K83, _:N84, _:G85, _:E86, _:V87, _:A88, _:A89, _:T90, _:K91, _:V92, _:G93, _:A94, _:L95, _:S96, _:K97, _:G98, _:Q99, _:L100, _:K101	52	0.73
_:W229, _:N230, _:Q231, _:A232, _:A233, _:L234, _:A235, _:M236, _:E237, _:G238, _:G239, _:G240, _:G241, _:S242, _:A243, _:Y244, _:T245, _:K246, _:L267, _:Q268, _:S269, _:L270, _:G271, _:A272, _:E273, _:I274, _:A275, _:V276, _:E277, _:H278, _:H279, _:H280, _:H281, _:H282, _:H283	35	0.71
_:K4, _:I5, _:I6, _:T9, _:D10, _:S12, _:F13, _:D14, _:T15, _:D16, _:V17, _:L18, _:K19, _:A20, _:D21, _:G22, _:F28, _:A30, _:E31, _:W32, _:C33, _:P35, _:C36, _:I39, _:I42, _:L43, _:I46, _:A47, _:D48, _:E49, _:H117	31	0.67

### 3.6 Immune stimulation of innate immune cells by the MP3RT vaccine

**Figure 5:**
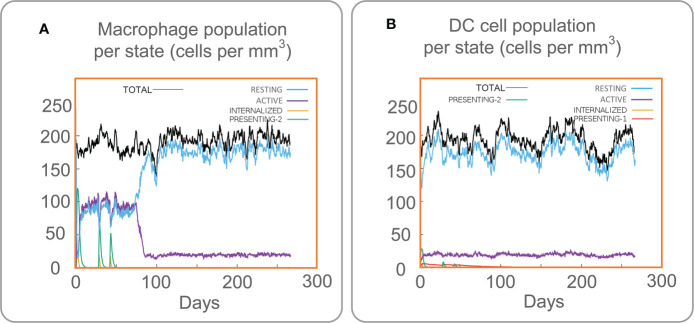
Immune stimulation of innate immune cells predicted by the C-ImmSim Server. **(A)** The macrophage population per state. **(B)** The DCs population per state.

The C-ImmSim server is a computer simulation tool. The changes in human immune cells can be observed by simulating the vaccine injection process through the C-ImmSim server. The C-ImmSim server results showed that the MP3RT vaccine could significantly activate macrophages (**Figure 5A**). The results suggested that the active macrophage population per state was maintained at 100 cells/mm^3^ from the first to the third injection. Eighty days after the first vaccination, the active macrophage population per state decreased rapidly and maintained at 20 cells/mm^3^. In addition, the presenting-2 macrophage population per state peaked three times, at 120 cells/mm^3^ on day 2, 70 cells/mm^3^ on day 28, and 50 cells/mm^3^ on day 44 after the first immunization. Moreover, dendritic cells (DCs) are the most robust antigen-presenting cells (APCs) and play an essential role in presenting vaccines to T cells (Cheng et al., 2022). Therefore, it was found that MP3RT vaccination could remarkably activate DCs (**Figure 5B**). After the stimulation of the MP3RT vaccine, the population per state of total DCs was maintained at 200 cells/mm^3^, and the population per state of active DCs rapidly secreted at 20 cells/mm^3^. Like macrophages, present-2 DCs also formed three peaks after three MP3RT stimulation.

### 3.7 Immune stimulation of adaptive immune cells by the MP3RT vaccine

**Figure 6:**
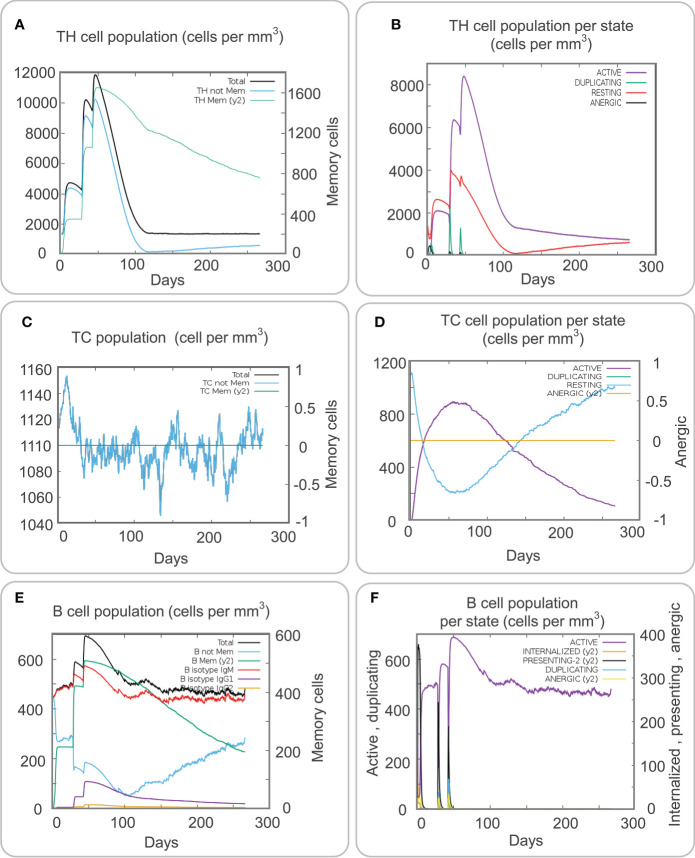
Immune stimulation of adaptive immune cells predicted by the C-ImmSim Server. **(A)** The TH cell population. **(B)** The TH cell population per state. **(C)** The Tc cell population. **(D)** The Tc cell population per state. **(E)** The B cell population. **(F)** The B cell population per state.

Adaptive immune cells, which play a significant role in resisting the pathogenesis of TB, mainly include CD4^+^ T cells, CD8^+^ T cells, and B cells. The traditional concept believes that B cells play an auxiliary role in killing *M. tuberculosis*, but recent studies have shown that the killing effect of humoral immunity generated by B cells on *M. tuberculosis* cannot be ignored (Gong et al., 2021a; Gong et al., 2022). Our results showed that the population per state of total, non-memory, and memory TH cells formed three higher and higher peaks after the MP3RT vaccine stimulation and finally reached the highest peak after the third stimulation (**Figure 6A**, 12000 cells/mm^3^, 10000 cells/mm^3^, and 11000 cells/mm^3^, respectively). At the same time, after the third stimulation, the population per state of active and resting CD4^+^ T cells reached peaks of 8500 cells/mm^3^ and 4000 cells/mm^3^, respectively (**Figure 6B**). Interestingly, the population per state of duplicating cells also reached three peaks, and the highest peak with 2200 cells/mm^3^ was observed after the second stimulation (**Figure 6B**).

Cytotoxic T lymphocytes can clear *M. tuberculosis* by producing perforin, granzyme B, and other cytotoxic factors. The results of the C-ImmSim server showed that the population per state of non-memory cytotoxic T lymphocytes formed a peak after three stimulations, and the highest value was 1150 cells/mm^3^ (**Figure 6C**). However, when the population per state of active cytotoxic T lymphocytes reached the peak (900 cells/mm^3^) on the 50th day after primary immunization, and the population per state of duplicating cytotoxic T lymphocytes reached the valley bottom (200 cells/mm^3^) on the contrary (**Figure 6D**). We also predicted the population per state of B cells after the MP3RT vaccine immunization. The results showed that the population per state of total B cells reached a peak of 690 cells/mm^3^ after the third stimulation (**Figure 6E**). In addition, the population per state of active B cells reached the peak (690 cells/mm^3^) after the third stimulation, and the population per state of presenting-2 cells reached the peak (650 cells/mm^3^) after the first stimulation (**Figure 6F**).

### 3.8 *In silico* and *in vivo* comparison of humoral immune responses induced by the MP3RT vaccine

**Figure 7:**
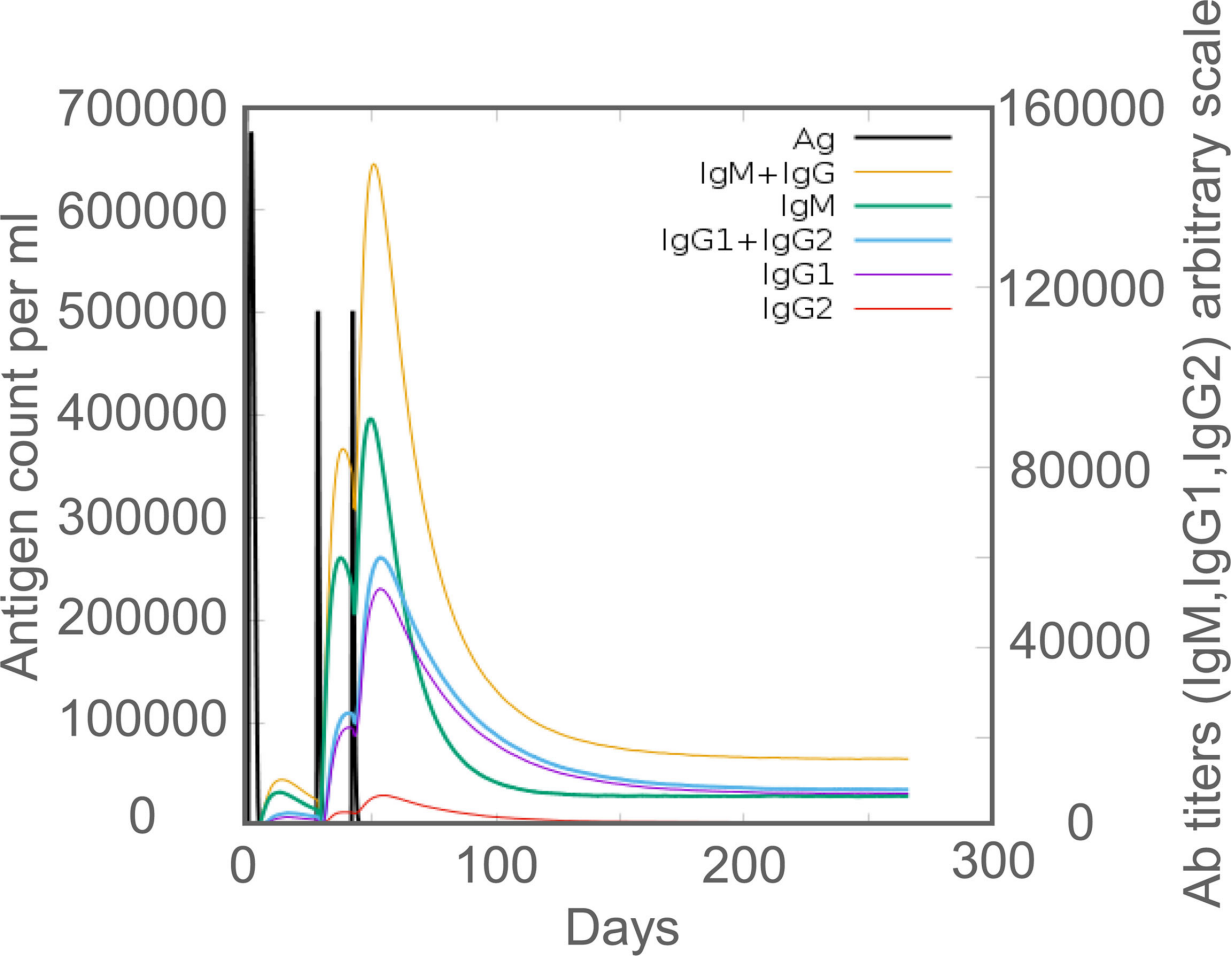
The levels of MP3RT-specific IgG, IgM, IgG1, and IgG2 antibodies predicted by the C-ImmSim server.

**Figure 8:**
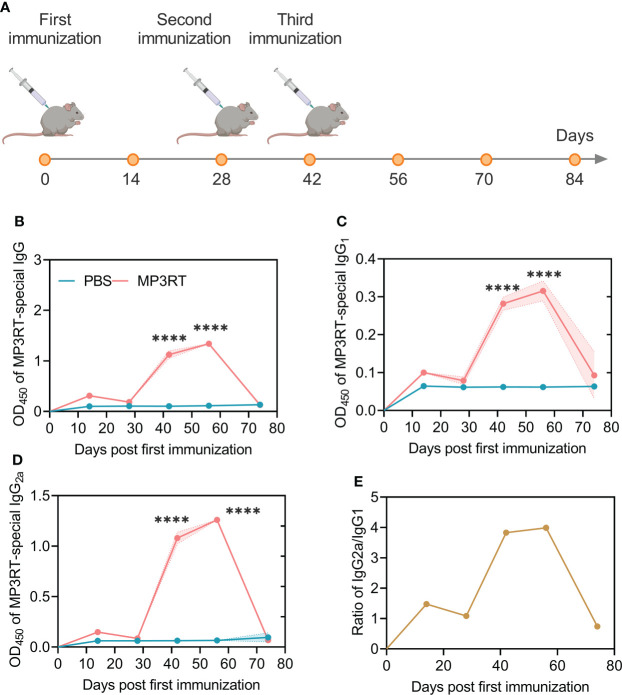
Schematic diagram of mouse immunization and detection of MP3RT-specific antibodies in mice. **(A)** Time of injection of the MP3RT vaccine and PBS in humanized mice. **(B–E)** The levels of the MP3RT-specific IgG, IgG_1_, IgG_2a_ antibodies, and IgG_2a_/IgG_1_ ratio in mice vaccinated with MP3RT and PBS. Six serum samples in PBS group or 7 serum samples in MP3RT group were pooled together and tested in quadruplicate for antibody detection. The results were analyzed with the Unpaired t-test or Mann-Whitney test according to the normality. All data were shown as mean + SEM (*n* = 4). *P* < 0.05 was considered significantly different. ****, *P* < 0.0001.

*In silico* analysis, it was found that MP3RT immunization produced significantly higher levels of MP3RT-specific antibodies. The level of IgM antibody reached the highest peak (400000ml) after the third injection, and then gradually decreased (**Figure 7**). The level of IgG1 + IgG2 antibodies reached the highest peak (650,000/ml) after the third immunization and then gradually decreased (**Figure 7**). Furthermore, the levels of IgG1 and IgG2 were 250,000/ml and 230,000/ml, respectively (**Figure 7**). *In vivo* analysis, we performed animal experiments to compare the bioinformatics-predicted immunological profiles with real-world experimental data of the MP3RT vaccine. The humanized mice were vaccinated on days 0, 28, and 42, respectively (**Figure 8A**). The results showed that the levels of MP3RT-specific IgG (**Figure 8B**), IgG1 (**Figure 8C**), and IgG2a (**Figure 8D**) in mice immunized with MP3RT were significantly higher than those in mice immunized with PBS. We also found that the ratio of IgG2a/IgG1 was more elevated than one after the first immunization (**Figure 8E**). A ratio of IgG2a/IgG1 >1 indicates a Th1-type immune response induced by the MP3RT vaccine, otherwise, a Th2-type immune response.

### 3.9 *In silico* and *in vivo* comparison of cellular immune responses induced by the MP3RT vaccine

**Figure 9:**
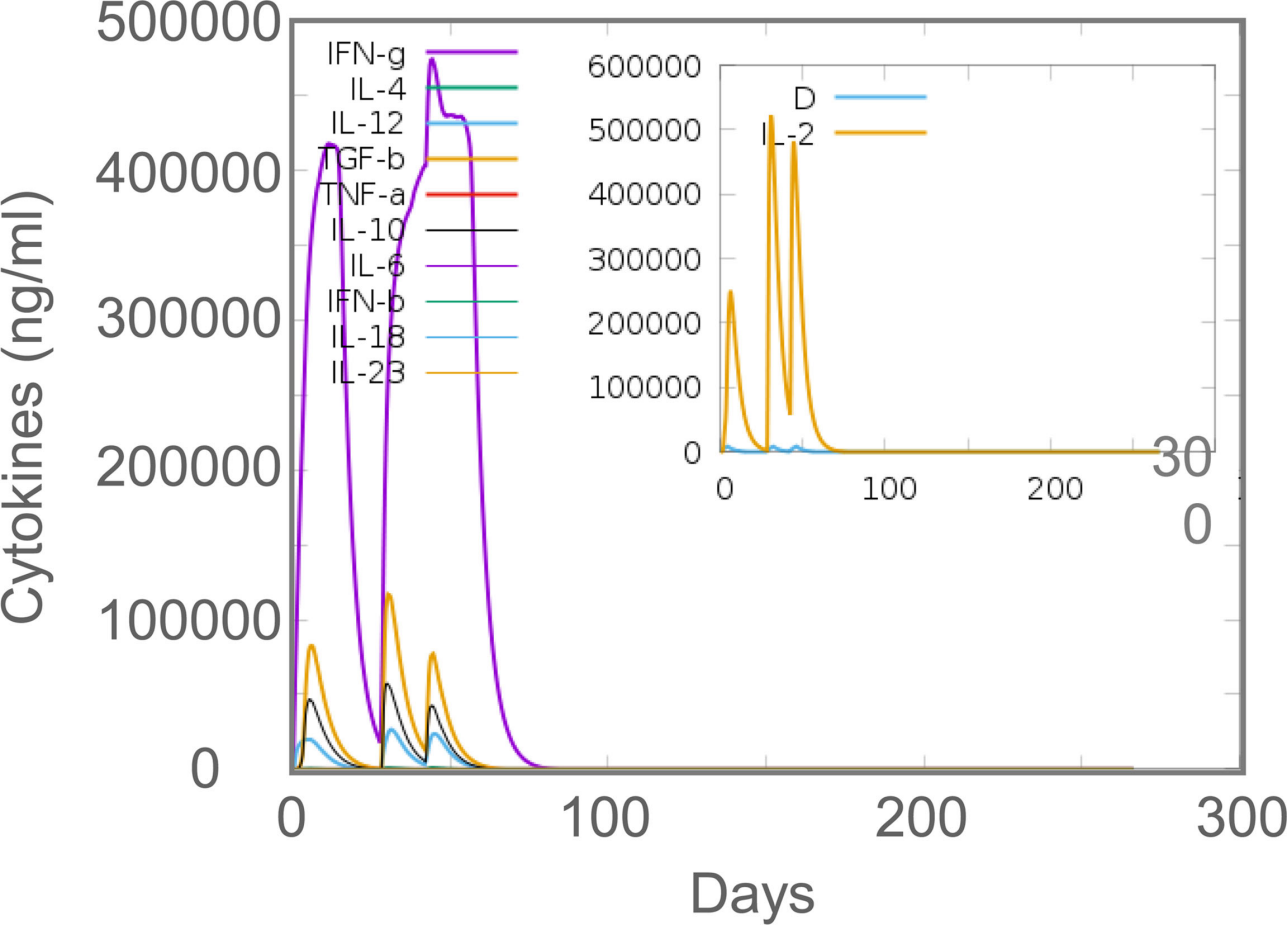
The levels of cytokines predicted by the C-ImmSim server.

**Figure 10:**
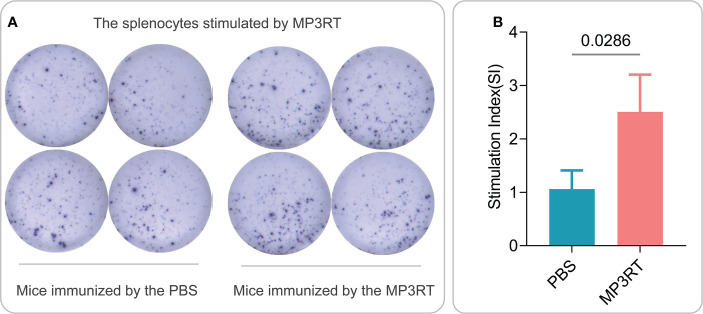
IFN-γ^+^ T lymphocytes detection with ELISPOT in mice. **(A)** The splenocytes collected from mice immunized with PBS or MP3RT vaccine were stimulated with MP3RT *in vitro*, and the SFCs were detected using a mouse ELISPOT kit. **(B)** The number of IFN-γ^+^ T lymphocytes (showed as SI) stimulated by the MP3RT vaccine in PBS and MP3RT groups were compared. In order to reduce individual differences, the spleens of mice in each group were mixed to prepare spleen cell suspensions, and then ELISPOT experiments were performed in quadruplicate. The results were analyzed with the Unpaired t-test or Mann-Whitney test according to the normality. All data were shown as mean + SEM (*n* = 4). *P* < 0.05 was considered significantly different.

**Figure 11:**
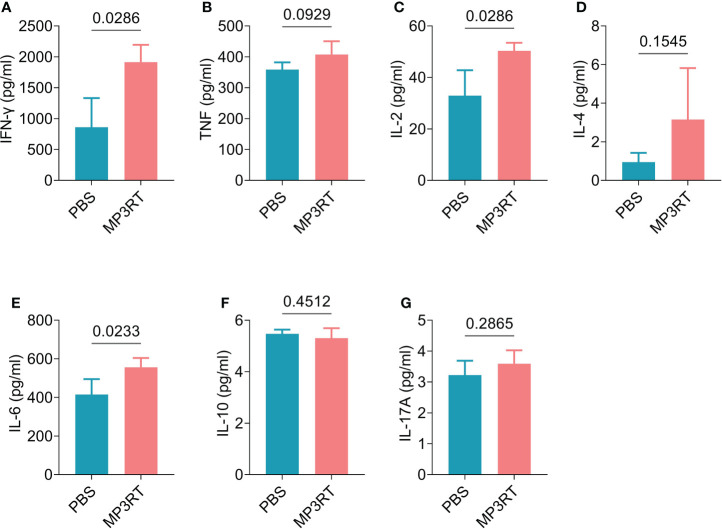
The levels of cytokines induced by the MP3RT vaccine in mice. The levels of IFN-γ **(A)**, TNF-α **(B)**, IL-2 **(C)**, IL-4 **(D)**, IL-6 **(E)**, IL-10 **(F)**, and IL-17A **(G)** cytokines induced by the MP3RT vaccine in the PBS group and MP3RT group were detected by a Mouse Th1/Th2/Th17 Cytokine Kit. In order to reduce individual differences, the spleens of mice in each group were mixed to prepare spleen cell suspensions, and then cytokine detection were performed in quadruplicate. The results were analyzed with the Unpaired t-test or Mann-Whitney test according to the normality. All data were shown as mean + SEM (*n* = 4). *P* < 0.05 was considered significantly different.

*In silico* analysis, the results showed that MP3RT vaccination could induce a significantly higher level of IFN-γ. The level of IFN-γ formed two peaks and reached the highest value in the second immunization (470000 ng/ml). Unlike the population of immune cells, the levels of cytokines such as IFN-γ, IL-2, TGF-β, IL-10, and IL-12 reached the highest peak after the second immune stimulation (**Figure 9**). *In vivo* analysis, ELISPOT results showed that the frequency of IFN-γ^+^ T lymphocytes in mice immunized with MP3RT was significantly higher than that in mice immunized with PBS (**Figure 10A**), and the SI value in the MP3RT group was substantially higher than that in PBS group (**Figure 10B**, *P*=0.0286). Additionally, we have explored the cytokines such as IFN-γ (**Figure 11A**), TNF-α (**Figure 11B**), IL-2 (**Figure 11C**), IL-4 (**Figure 11D**), IL-6 (**Figure 11E**), IL-10 (**Figure 11F**), and IL-17A (**Figure 11G**) induced by the MP3RT vaccine. We found that the levels of IFN-γ (*P* = 0.03), IL-2 (*P* = 0.03), and IL-6 (*P* = 0.02) in mice vaccinated with the MP3RT vaccine were significantly higher than those in PBS immunized mice.

## 4 Discussion

TB is a global public health problem, and its complex pathogenesis and emerging drug resistance seriously hinder WHO’s plan to end TB. As early as 2014, Wilkie, M. E. and McShane, H. conducted a study termed “The need for a blueprint to progress in vaccine development” to set the tone for the strategic direction of vaccine research for the next decade (Wilkie and McShane, 2015). They have established innovative mechanisms to reduce global TB incidence in five key fields, one of which is the development of new TB vaccines by identifying correlations between immunity and biomarkers. Peptide-based vaccines belong to subunit vaccines, which have attracted widespread attention with their remarkable advantages. In particular, the rapid development of bioinformatics technologies has given wings to the development of peptide-based vaccines (Wang et al., 2017; Gong et al., 2021a; Gong et al., 2021b; Gong et al., 2022). Furthermore, applying computational tools and immunoinformatic techniques saves a lot of workforce and material resources for vaccine research and development (Rapin et al., 2010; Moodley et al., 2022).

Our previous study constructed a peptide-based vaccine, MP3RT, and found that the MP3RT vaccine showed good immunogenicity, antigenicity, and significant protection against *M. tuberculosis* H37Rv strain infection in humanized mice (Gong et al., 2021a). Interestingly, further study showed that the protective mechanism of the MP3RT vaccine depends on the activated CD3^+^CD4^+^ T lymphocytes and CD3^+^IFN-γ^+^ T lymphocytes as well as high levels of IFN-γ and IgG antibodies (Gong et al., 2021a). Unfortunately, this previous study only focused on the immunological characteristics and protective mechanism of the MP3RT vaccine in animal models but did not analyze the consistency between bioinformatics prediction results and animal experimental results. Hence, this study was performed to bridge this gap.

Herein, we first predicted the physicochemical parameters, secondary structure, tertiary structure, and immune stimulation of innate/adaptive immune cells induced by the MP3RT vaccine using computational tools and immunoinformatic techniques. We found that: 1) MP3RT is a non-sensitizing and non-toxic hydrophilic vaccine, and its antigenicity and immunogenicity were 0.88 and 0.61, respectively. The good antigenicity and immunogenicity of the MP3RT vaccine lay the foundation for good protective efficiency. 2) The instability index, fat index, and solubility of the MP3RT vaccine were 29.65, 76.08, and 0.55, indicating that this vaccine was not easily degraded and was water-soluble. 3) α-helix accounted for 53.36% of the secondary structure of the MP3RT vaccine, which increased the recognition between MP3RT-specific antibodies and the MP3RT vaccine and promoted a more robust immune response (Corradin et al., 2007). 4) The Ramachandran plot analysis of the 3D model of the MP3RT vaccine found that the Favored region accounted for 98.22%, indicating that the model was of good quality and could be used for molecular docking. When the MP3RT vaccine was docked with the HLA-DRB1*01:01, TLR-2, and TLR-4 receptors, the binding affinities were -1234.10 kcal/mol, -1066.40 kcal/mol, and -1250.40 kcal/mol, suggesting that the binding between the MP3RT vaccine and these three receptors was stable.

These results revealed that the MP3RT vaccine might stimulate immune responses based on its high antigenicity and immunogenicity, stable structure, and high affinity. To further clarify the immunoinformatics characteristics of the MP3RT vaccine, we used C-IMMsim Server to simulate the cellular immune response induced by the MP3RT vaccine. The cellular immune simulation results suggested that the active macrophage population per state was maintained at 100 cells/mm^3^ from the first to the third injection, the population per state of total DCs was maintained at 200 cells/mm^3^, and the population per state of active DCs rapidly secreted at 20 cells/mm^3^. These data indicated that the MP3RT vaccine could cause innate immune responses by activating macrophages and DC cells. Macrophages are susceptible to regulatory factors *in vivo*. They can express high levels of IL-10 and IFN-γ receptors and bind with IFN-γ from CD4^+^T cells to regulate the production of cytokines such as IL-1α, IL-1β, and TNF-α *in vivo* and inhibit the growth of *M. tuberculosis* (Mayer-Barber et al., 2011). In addition, DC is the most robust APC, which can activate immature T cells to produce an adaptive immune response and migrate to draining lymph nodes (Leepiyasakulchai et al., 2013). These data demonstrated that these activated APCs could trigger adaptive immunity and bridge innate and adaptive immunities, confirmed by our following immune stimulation prediction. Precited results of the C-ImmSim server found that three doses of immunization with the MP3RT vaccine significantly activated CD4^+^ T cells to produce high levels of IFN-γ and TNF-α cytokines to kill *M. tuberculosis* (Mayer-Barber and Barber, 2015). Green, A. M. et al. observed that RAG KO mice reconstructed with a mixture of IFN-γ KO CD4^+^ T cells and WT CD4 depleted spleen cells died earlier than RAG KO mice rebuilt with WT CD4^+^ T cells and WT CD4 depleted spleen cells after infected with *M. tuberculosis*, demonstrating the critical role of IFN-γ^+^ CD4^+^ T cells in fighting against *M. tuberculosis* infection (Green et al., 2013). The results from ELISPOT showed that the MP3RT vaccine stimulated a high frequency of IFN-γ^+^ T lymphocytes not only in MP3RT-immunized mice but also in PBS-immunized mice. Interestingly, these predicted results were consistent with the results from ELISPOT in humanized mice in this study and our previous study (Gong et al., 2021a). Results from cytokines detection also indicated that the MP3RT vaccine could stimulate high levels of INF-γ and IL-2 cytokines in MP3RT- and PBS-immunized mice. Interestingly, immunoinformatics prediction results showed that the MP3RT could induce significantly high levels of IFN-γ and IL-2. Together, these data demonstrate that the MP3RT vaccine could generate high cellular immune responses and provide new evidence to assess the consistency between immunoinformatics predictions and real-world experimental results.

The cellular immune response is critical in host resistance to *M. tuberculosis*. However, more and more evidence indicates that the humoral immune response also plays an essential role in killing *M. tuberculosis* (Bitencourt et al., 2021; Gong et al., 2022; Nziza et al., 2022). This study explored the humoral immunity induced by the MP3RT vaccine. Our animal experiment showed that after immunizing the humanized mice with three doses of the MP3RT vaccine, the levels of MP3RT-specific IgG, IgG1, and IgG2a were significantly higher than those in the control group. Furthermore, the levels peaked on the 56th day after the first immunization, indicating that the MP3RT vaccine can stimulate B lymphocytes to produce significantly high levels of MP3RT-specific antibodies. Coincidentally, the results of these animal experiments are consistent with the results predicted by our immunoinformatics technology. In our immunoinformatics prediction, we found that the MP3RT vaccine induced high levels of IgM, IgG1, and IgG2 antibodies. Furthermore, these antibodies peaked at a time similar to real animal experiments. As part of humoral immunity, antibodies have been found to increase macrophage responses to intracellular *M. tuberculosis* (such as phagolysosomal maturation and pyrophosphorylation-independent inflammasome activation) to control the growth of *M. tuberculosis* (Lu et al., 2016). It was also found in animal experiments that mice lacking the ability to secrete antibodies were more susceptible to *M. tuberculosis* infection (Torrado et al., 2013). A *post-hoc* analysis of the MVA85A vaccine also determined that elevated Ag85A-specific antibody titers were associated with lower TB risk (Fletcher et al., 2016). This evidence indicates that antibodies play an important role in fighting against *M. tuberculosis* infection. Therefore, the high levels of antibodies induced by the MP3RT vaccine were beneficial in inhibiting the growth of *M. tuberculosis*.

This study has some limitations: (1) MP3RT vaccines consist only of HTL epitopes and lack CTL epitopes and B-cell epitopes, which may reduce the effectiveness of the immune response. (2) Not all the predictions were verified by wet experiments, such as CD4+T cells, CD8+T cells, IL-12, etc. (3) Although the level of IgG1 antibody was higher than that of the IgG2 antibody in bioinformatics prediction, but the level of IgG2a antibody was higher than that of IgG1 antibody in animal experiments, and we still need to conduct more in-depth research to verify this result.

## 5 Conclusion

In summary, computational tools predicted that MP3RT was a non-toxic and sensitizing peptide-based vaccine with high antigenicity and immunogenicity. Immunoinformatics prediction showed that the MP3RT vaccine bound stably to the HLA-DRB1*01:01, TLR-2, and TLR-4 receptors and could effectively stimulate APCs such as macrophages and DCs to present peptides, thereby activating T lymphocytes and B lymphocytes to produce high levels of cytokines such as IFN-γ and IL-2 and antibodies. Real-world animal experimental data revealed that the MP3RT vaccine significantly increased IFN-γ^+^ T lymphocytes to secrete Th1-type cytokines such as IFN-γ, IL-2, and IL-6, and stimulated B lymphocytes to produce high levels of MP3RT-specific antibodies. Our study demonstrated that bioinformatics and immunoinformatics tools had exhibited excellent performance in predicting the physicochemical properties, molecular structure, and immunological characteristics of peptide-based vaccines, and the results of humoral and cellular immune responses predicted by the computational tool are in high consistent with real-world animal experimental data, suggesting that immunoinformatic techniques can be generalized in the field of reverse vaccinology.

## Data availability statement

The datasets presented in this study can be found in online repositories. The names of the repository/repositories and accession number(s) can be found in the article/**Supplementary Material**.

## Ethics statement

Protocols on mice were approved by the Animal Ethical Committee of the 8th Medical Center of PLA General Hospital (Approved Number: 309201808171015).

## Author contributions

Conceptualization: WG and LW; Data curation: PC and YX; Formal analysis: PC and YX; Funding acquisition: WG; Methodology: PC, YX, JW, ZJ, and WG; Software: PC, YX, and WG; Writing - original draft: PC; Writing - review & editing: WG and LW. All authors contributed to the article and approved the submitted version.

## Funding

This study was funded by the Beijing Municipal Science & Technology Commission (Grant No. 19L2065 and 7212103).

## Conflict of interest

The authors declare that the research was conducted in the absence of any commercial or financial relationships that could be construed as a potential conflict of interest.

## Publisher’s note

All claims expressed in this article are solely those of the authors and do not necessarily represent those of their affiliated organizations, or those of the publisher, the editors and the reviewers. Any product that may be evaluated in this article, or claim that may be made by its manufacturer, is not guaranteed or endorsed by the publisher.
