# Supplementary material for: Evaluation of the consistence between the results of immunoinformatics predictions and real-world animal experiments of a new tuberculosis vaccine MP3RT
Source: Front Cell Infect Microbiol. 2022 Nov 2;12:1047306. doi: 10.3389/fcimb.2022.1047306 (PMC9666678; doi:10.3389/fcimb.2022.1047306)
Supplement: Supplementary file 1 [file Table_1.doc]

| Ligand amino acid | Distance | Acceptor amino acid | Ligand amino acid | Distance | Acceptor amino acid |
| --- | --- | --- | --- | --- | --- |
| LEU-18 | 1.7 | ARG-123 | GLY-125 | 2.3 | GLU-187 |
| LYS-19 | 1.8 | ARG-123 | LYS-140 | 1.7 | THR-172 |
| ASP-21 | 1.9 | ARG-100 | LYS-140 | 1.8 | GIU-187 |
| ASP-21 | 2.2 | ARG-100 | LYS-140 | 2.7 | GIU-187 |
| ASP-21 | 2.2 | ARG-100 | GLU-142 | 2.3 | GLN-174 |
| ASP-48 | 1.8 | LYS-105 | GLY-179 | 2.3 | GLU-101 |
| ASP-48 | 2.2 | GLN-107 | ASP-223 | 1.9 | ARG-50 |
| ASP-105 | 2.0 | ARG-191 | LYS-246 | 1.9 | GLU-87 |
| ASP-105 | 1.9 | ARG-191 | LYS-246 | 2.1 | GLY-84 |
| ASN-107 | 1.9 | ARG-191 | ARG-253 | 1.8 | ARG-94 |
| MET-116 | 2.0 | GLU-98 | ARG-253 | 1.9 | GLU-87 |
| HIS-117 | 2.0 | GLU-98 | ARG-253 | 2.7 | SER-88 |
| HIS-122 | 2.0 | GLU-98 | ASP-256 | 1.8 | ARG-94 |
| SER-123 | 2.0 | GLU-187 | ASP-265 | 2.1 | ARG-94 |
| SER-123 | 2.3 | VAL-101 |  |  |  |
