# Supplementary material for: Evaluation of the consistence between the results of immunoinformatics predictions and real-world animal experiments of a new tuberculosis vaccine MP3RT
Source: Front Cell Infect Microbiol. 2022 Nov 2;12:1047306. doi: 10.3389/fcimb.2022.1047306 (PMC9666678; doi:10.3389/fcimb.2022.1047306)
Supplement: Supplementary file 2 [file Table_2.doc]

| Ligand amino acid | Distance | Acceptor amino acid | Ligand amino acid | Distance | Acceptor amino acid |
| --- | --- | --- | --- | --- | --- |
| HIS-145 | 1.9 | GLN-198 | GLU-237 | 1.9 | ARG-486 |
| GLY-152 | 1.9 | THR-174 | GLY-238 | 1.7 | LYS-422 |
| GLY-152 | 2.1 | GLY-172 | SER-242 | 1.7 | LYS-422 |
| ASP-154 | 1.8 | LYS-150 | LYS-246 | 1.8 | GLU-369 |
| ASP-154 | 1.9 | LYS-150 | LYS-246 | 1.9 | GLU-344 |
| ASP-154 | 1.9 | THR-149 | TRP-249 | 1.9 | TYR-364 |
| ASP-155 | 2.0 | PHE-175 | ARG-253 | 1.7 | GLU-281 |
| LYS-158 | 1.8 | THR-149 | ARG-253 | 2.4 | GLU-281 |
| LYS-158 | 2.5 | LEU-151 | GLN-268 | 1.9 | LYS-567 |
| TYR-209 | 1.8 | GLN-526 | SER-269 | 1.7 | LYS-567 |
| TYR-209 | 2.0 | GLN-526 | GLU-273 | 1.8 | LYS-527 |
| TYR-224 | 1.7 | LYS-480 | GLU-273 | 1.8 | LYS-527 |
| ARG-227 | 1.8 | GLU-481 | GLU-277 | 1.8 | LYS-505 |
| ARG-227 | 1.9 | GLU-460 | GLU-277 | 1.8 | ARG-508 |
| ASN-230 | 1.9 | TYR-440 | GLU-277 | 1.8 | ARG-508 |
| GLN-231 | 1.8 | LYS-505 | GLU-277 | 2.1 | TRP-529 |
| LEU-234 | 1.9 | ARG-486 | HIS-281 | 1.9 | SER-559 |
| ALA-235 | 1.7 | ARG-486 | HIS-282 | 1.7 | ARG-508 |
| GLU-237 | 1.8 | ARG-508 | HIS-282 | 2.3 | ARG-508 |
| GLU-237 | 2.0 | ARG-508 | HIS-283 | 1.8 | ARG-508 |
| GLU-237 | 1.9 | ARG-486 |  |  |  |
