# Supplementary material for: Evaluation of the consistence between the results of immunoinformatics predictions and real-world animal experiments of a new tuberculosis vaccine MP3RT
Source: Front Cell Infect Microbiol. 2022 Nov 2;12:1047306. doi: 10.3389/fcimb.2022.1047306 (PMC9666678; doi:10.3389/fcimb.2022.1047306)
Supplement: Supplementary file 3 [file Table_3.doc]

| Ligand amino acid | Distance | Acceptor amino acid | Ligand amino acid | Distance | Acceptor amino acid |
| --- | --- | --- | --- | --- | --- |
| SER-2 | 2.0 | GLU-144 | TRP-29 | 1.8 | LYS-39 |
| LYS-4 | 1.7 | GLU-136 | TRP-29 | 1.9 | CYS-37 |
| LYS-4 | 1.9 | GLU-136 | GLU-31 | 2.2 | GLN-41 |
| ASP-10 | 1.8 | LYS-89 | LYS-134 | 1.7 | GLU-605 |
| ASP-10 | 2.1 | TYR-79 | LYS-134 | 1.9 | GLU-605 |
| ASP-10 | 2.6 | TYR-79 | GLU-142 | 2.0 | GLN-599 |
| ASP-14 | 2.0 | LYS-89 | GLU-142 | 2.1 | GLN-599 |
| ASP-14 | 1.9 | GLN-484 | THR-213 | 1.7 | LYS-91 |
| ASP-14 | 2.7 | GLN-484 | THR-213 | 2.0 | GLU-143 |
| ASP-16 | 1.9 | ASN-464 | THR-213 | 2.6 | GLU-143 |
| ASP-16 | 1.9 | ASN-464 | ASP-223 | 2.2 | ARG-382 |
| ASP-16 | 1.9 | ASN-464 | ASN-230 | 2.0 | TYR-403 |
| ASP-16 | 2.1 | ASN-86 | SER-242 | 1.9 | GLU-474 |
| VAL-17 | 2.1 | GLN-484 | LYS-246 | 1.7 | GLU-474 |
| LYS-19 | 1.7 | ASN-464 | LYS-246 | 1.8 | GLU-474 |
| LYS-19 | 1.7 | ASP-490 | ARG-253 | 2.5 | THR-499 |
| ASP-27 | 1.7 | LYS-39 | ARG-253 | 1.8 | GLN-523 |
| ASP-27 | 1.9 | LYS-39 | ARG-253 | 2.0 | GLN-523 |
| PHE-28 | 1.8 | LYS-39 |  |  |  |
